# Pyogranulomatous pneumonia associated with *Actinomyces hyovaginalis* infection in a free-ranging Brazilian wild boar

**DOI:** 10.1007/s11259-026-11471-2

**Published:** 2026-08-22

**Authors:** Gabriela Hartmann, Diego Andrés Rodríguez Matarrita, Cassiane Elisabete Lopes, Joanna Vargas Zillig Echenique, Mônica Slaviero, Ana Paula Gobbi de Bitencourt, Franciele Maboni Siqueira, David Driemeier, Welden Panziera

**Affiliations:** 1 Setor de Patologia Veterinária, Universidade Federal do Rio Grande do Sul (UFRGS), Porto Alegre, Rio Grande do Sul (RS) Brazil; 2 Laboratório de Bacteriologia Veterinária, Universidade Federal do Rio Grande do Sul, Porto Alegre, RS Brazil; 3 Secretaria da Agricultura, Pecuária, Produção Sustentável e Irrigação (Seapi), Porto Alegre, RS Brazil; 4 Programa de Pós-Graduação em Ciências Veterinárias, Universidade Federal do Rio Grande do Sul, Porto Alegre, RS Brazil

**Keywords:** *Schaalia hyovaginalis*, Bacterial pneumonia, Actinomycota, Pulmonary actinomycosis, Wild hog

## Abstract

*Actinomyces (A.) hyovaginalis* is a Gram-positive, rod-shaped, polymorphic bacterium. In domestic pigs, the infection is typically associated with pyogranulomatous pneumonia. In wild boars, this infection was never associated with such lesion. We report the gross, histopathological, and bacteriological findings in a case of pyogranulomatous pneumonia caused by *A. hyovaginalis* infection in a free-ranging wild boar. Fragments of the lungs, lymph nodes, and heart from a wild boar were sent by boar hunters for histopathological analysis with a presumptive tuberculosis diagnosis. Grossly, in the lungs, there were multiple nodules of 0.1 to 0.8 cm in diameter, with light-yellow, opaque, friable necrotic centers. Histologically, these nodules were composed of coagulative necrosis, with abundant amorphous to granular eosinophilic material, neutrophilic inflammatory infiltrate, cellular debris, multifocal mineralization, and numerous intralesional extracellular coccobacillary basophilic bacterial colonies within the necrotic centers. Surrounding these areas, there were concentric layers of a marked inflammatory infiltrate of lymphocytes, plasma cells, macrophages, epithelioid macrophages, and neutrophils. The Brown-Hopps stain evidenced intralesional Gram-positive bacilli. In bacterial culture, small white partial hemolytic colonies were identified in 5% blood sheep agar. The isolated bacterium was identified as *A. hyovaginalis* by MALDI-TOF MS. Based on the gross, histopathological, and bacteriological findings, a diagnosis of pyogranulomatous pneumonia by *A. hyovaginalis* was established. Although not previously reported in this suid species, this infection should be considered a differential diagnosis for pyogranulomatous pulmonary lesions in wild boars.

## Background

*Actinomyces (A.) hyovaginalis*, formally proposed to be reclassified into the genus *Schaalia* (Alssahen et al. 2020), is a polymorphic, catalase-negative, rod-shaped, slow-growing, non-acid-fast, Gram-positive bacterium (Reichel and Wragg 2007; Broes et al. 2019). This agent was first isolated from purulent vaginal discharges of sows, aborted fetuses, and multiple tissue samples of domestic pigs (*Sus scrofa domesticus*) (Collins et al. 1993; Hommez et al. 1991; Wübbelmann et al. 2016). In domestic pigs, this infection is most commonly associated with sporadic disseminated pyogranulomatous lung lesions with extensive necrotic centers (Aalbæk et al. 2003; Reichel and Wragg 2007). In addition to pulmonary lesions in slaughtered domestic pigs, this agent has been also associated with caseous lymphadenitis (Foster et al. 2012), mastitis (Murakami et al. 1998; Christensen et al. 2007), abortion with necrotizing and suppurative placentitis in sows (Storms et al. 2002; Hogg et al. 2012), and suppurative bronchopneumonia with intralesional bacteria in aborted swine fetuses (Hogg et al. 2012). Moreover, osteomyelitis of the mandibular bone after tusk extraction was reported in a pot-bellied pig (Savard et al. 2021). In other animal species, *A. hyovaginalis* was isolated from caseous lymphadenitis and multiple other systemic pyemic lesions of sheep, goats, and moufflon (Schumacher et al. 2009; Foster et al. 2012; De La Fuente et al. 2017; Alssahen et al. 2020; Marinoff et al. 2020; Sting et al. 2020), abscesses in alpacas and llamas (Sting et al. 2020), and a case of suppurative arthritis in a captive zoo giraffe (Wickhorst et al. 2017).

In wild boars (*Sus scrofa*), *A. hyovaginalis* has previously been isolated from lung samples of an animal exhibiting severe lungworm infestation, fibrinopurulent bronchopneumonia, and pulmonary haemorrhage (Alssahen et al. 2020); however, in that case, *Escherichia coli*, *Staphylococcus chromogenes*, and α-hemolytic streptococci were also isolated from the same samples (Alssahen et al. 2020). Besides that, pyogranulomatous pneumonia caused directly by *A. hyovaginalis* infection is not described in this suid species. The main agents associated with bacterial pneumonia in wild boars are the same as those in domestic pigs, including *Mycoplasma hyopneumoniae*, *Pasteurella multocida*, *Streptococcus suis* serotype 2, *Actinobacillus pleuropneumoniae*, *Glaeserella parasuis*, and *M. hyorhinis* (Biondo et al. 2021; Souza et al. 2021; Silva Andrade et al. 2022; Ninkovic et al. 2024). However, none of these agents classically induces a pyogranulomatous or granulomatous reaction. In turn, this type of inflammation is occasionally induced by *Mycobacterium bovis* infection in both wild boars and domestic pigs (Zanella et al. 2008; Lopes et al. 2021a). Therefore, we report the gross, histological, and bacteriological findings in a case of pyogranulomatous pneumonia associated with *A. hyovaginalis* infection in a free-ranging wild boar as a differential diagnosis for bovine tuberculosis in this suid species.

### Case presentation

**Fig. 1:**
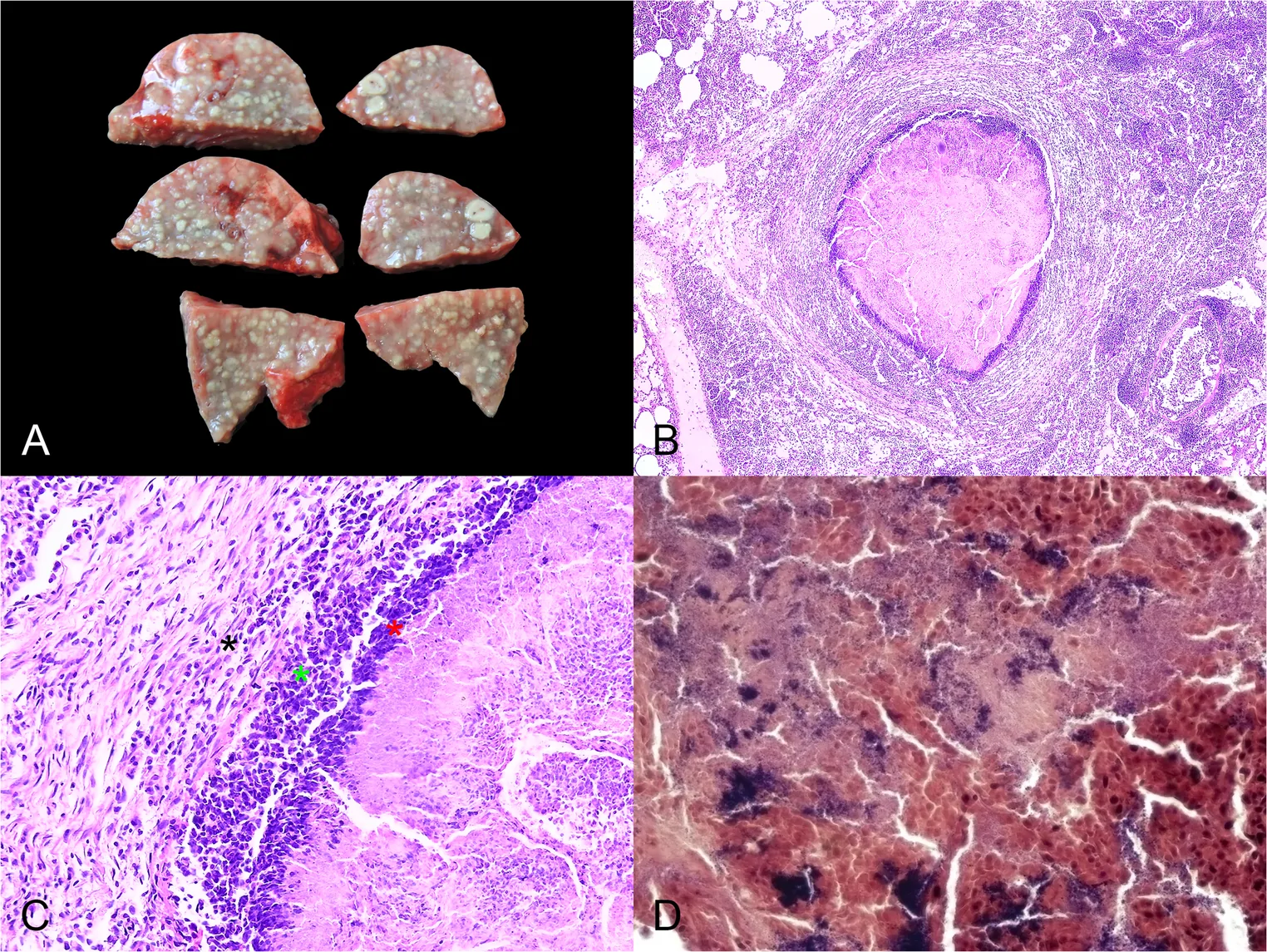
Pyogranulomatous pneumonia caused by *Actinomyces hyovaginalis* infection, wild boar. **A** In the lung sections, there were multiple 0.1–0.8 cm white nodules with light-yellow, friable necrotic centers. Bar, 2 cm. **B** Lung. A central pyogranuloma surrounded by a marked neutrophilic inflammatory infiltrate within the alveolar spaces and bronchiolar lumen. Hematoxylin and eosin (HE). Bar, 500 μm. **C** Lung. Close-up view of the pyogranuloma. A moderate inflammatory infiltrate composed of lymphocytes, plasma cells (black asterisk), and macrophages (green asterisk), and mild fibrous tissue deposition surrounded a central necrotic area with degenerate neutrophils (red asterisk). HE. Bar, 150 μm. **D** Lung. Multiple intralesional bacterial colonies within the necrotic center. Brown-Hopps (Gram) stain. Bar, 50 μm

Sections of the lungs, heart, and an unspecified intrathoracic lymph node from a free-ranging adult wild boar were submitted by wild boar hunters to the Official Veterinary Service of Rio Grande do Sul state, Brazil, due to suspected tuberculosis. The tissue samples were sent to the Veterinary Pathology Section of the Universidade Federal do Rio Grande do Sul for further evaluation. Grossly, the lung samples contained multiple, often coalescing, 0.1 to 0.8 cm-diameter nodules filled with light-yellow, opaque, friable material **(**Fig. 1A**)**. The remaining lung parenchyma was multifocally pale and firm, and failed to collapse. There were no gross lesions in the lymph node and heart, and, according to the hunters, no other lesions were observed in the other organs. Sections of the lungs, heart, and lymph node were collected and fixed in 10% neutral-buffered formalin. After fixation, the tissue samples were routinely processed and embedded in paraffin for histopathological evaluation. Three-µm-thick sections were stained with hematoxylin and eosin (HE), Brown-Hopps (modified Gram), Ziehl-Neelsen, and Grocott-Gomori methenamine silver (GMS) stains. Additionally, a lung sample stored at 4 °C for 4 h was submitted to bacteriological culture. The sample was plated on 5% sheep blood agar and MacConkey agar, and incubated at 37 °C under aerobic and microaerophilic conditions for 48 h. Bacterial identification was performed by Matrix-Assisted Laser Desorption Ionization-Time of Flight Mass Spectrometry (MALDI-TOF MS).

Histologically, in the lung parenchyma, there were multiple areas of coagulative necrosis **(**Fig. 1B**)**, with abundant amorphous to granular eosinophilic material, multifocal inflammatory infiltrate composed of degenerate neutrophils, cellular debris, multifocal deposition of coarsely granular, intensely basophilic material (mineralization), and numerous intralesional 2–3 μm coccobacillary basophilic bacterial aggregates. Surrounding these areas, there was a marked concentric inflammatory infiltrate of lymphocytes, plasma cells, macrophages, epithelioid macrophages, and neutrophils **(**Fig. 1C**)**. Occasionally, the macrophages palisade at the periphery. These necrotic areas were encircled by a moderate fibrous tissue proliferation. No Splendore-Hoeppli reaction was seen. The adjacent lung parenchyma showed numerous alveolar macrophages, marked multifocal neutrophilic inflammatory infiltrate, and fibrin-rich alveolar edema within the alveoli. No significant microscopic lesions were seen in the lymph node and heart sections. Brown-Hopps staining identified the intralesional coccobacilli as Gram-positive **(**Fig. 1D**)**. Moreover, no acid-fast bacilli nor fungal structures were identified within the lungs, lymph nodes, or heart by the Ziehl-Neelsen and GMS stains, respectively.

**Fig. 2:**
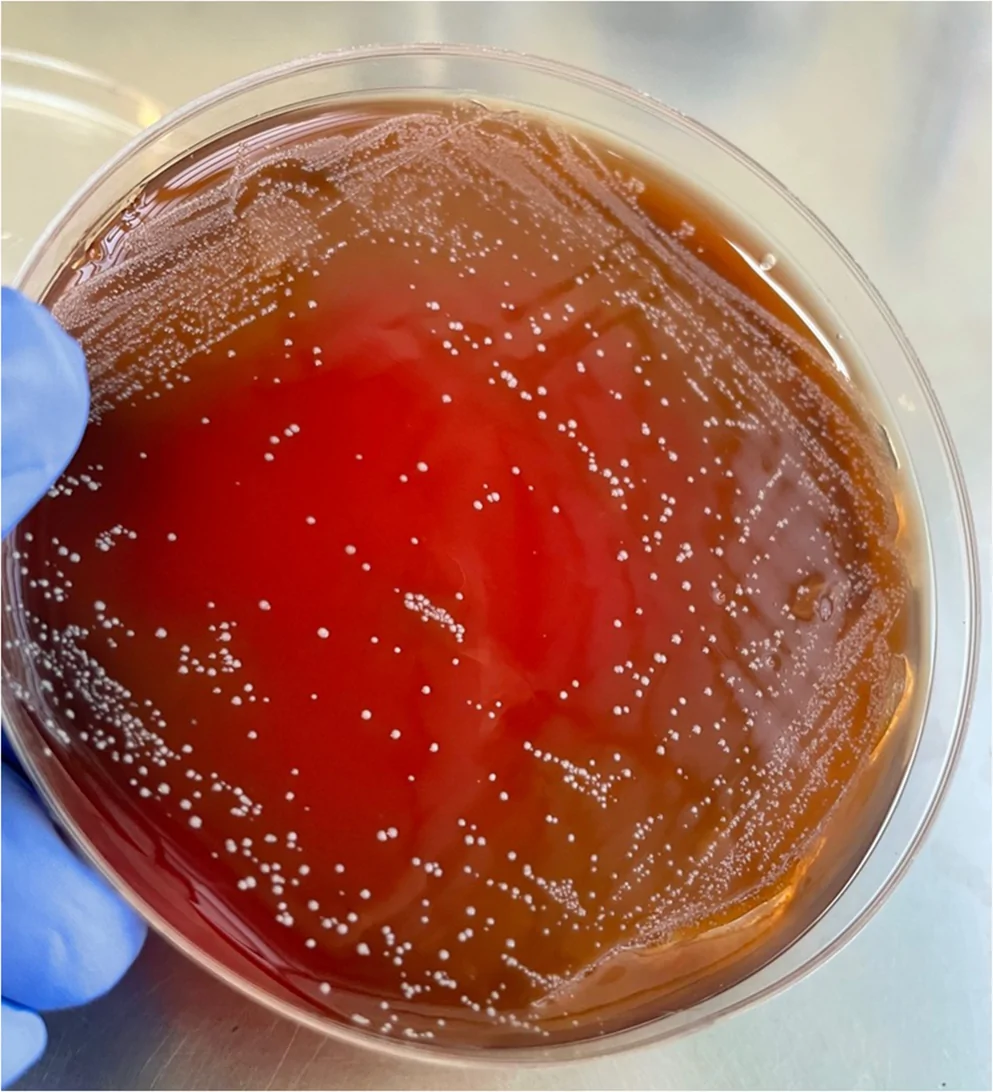
Bacterial culture of the *Actinomyces hyovaginalis* strain isolated in the research. White colonies with partial hemolysis after 48 h of incubation

After 48 h of bacteriological incubation in aerobiosis and microaerobiosis, small, white, partial-hemolytic pure colonies were visualized in 5% sheep blood agar **(**Fig. 2**)**, in both atmospheres. No growth was detected in MacConkey agar. Gram-positive pleomorphic coccobacilli were identified at the Gram stain. The isolated bacterium was identified as *A. hyovaginalis* by MALDI-TOF MS with an identity score of 2.6.

## Discussion

Based on the gross, histopathological, and bacteriological findings, a diagnosis of pyogranulomatous pneumonia by *A. hyovaginalis* was established. In our search, we found no histopathological descriptions of pyogranulomatous pneumonia associated with *A. hyovaginalis* in wild boars. A similar pyogranulomatous pattern with necrotic centers, similar to the pattern observed in the present case, is occasionally induced by *A. hyovaginalis* infection in domestic pigs (Liljegren et al. 2003; Aalbæk et al. 2003; Reichel and Wragg 2007). Moreover, bronchiolar ectasia is rarely associated with this infection in domestic pigs (Liljegren et al. 2003), but this finding was not present in the lung sections of the boar.

As in many countries worldwide, wild boars are one of the most important invasive animals in Brazil, and boar hunting has been permitted in the country since 2013 as a population control measure (Rosa et al. 2018). This suid species can harbor multiple pathogens, posing a threat to commercial pig farming, other livestock, humans, and native biodiversity (Rosa et al. 2018; Lopes et al. 2021b; Silva Andrade et al. 2022; Kmetiuk et al. 2023). Although the commercialization of boar meat and by-products is prohibited, their consumption and handling by hunters are allowed (Kmetiuk et al. 2023). Moreover, wild boars may serve as reservoirs for several porcine pathogens, including important respiratory agents, such as *Mycoplasma hyopneumoniae* (Biondo et al. 2021; Souza et al. 2021; Ninkovic et al. 2024). Therefore, when lesions are identified in carcasses of wild boars, it is essential that hunters submit tissue samples for histopathological and microbiological analysis, as was done in the present case, given the importance of identifying and reporting circulating agents in this population.

The source of *A. hyovaginalis* infection in domestic pigs remains unknown (Aalbæk et al. 2003; Schumacher et al. 2009). Some strains were isolated from uterine cervix swabs of healthy gilts and sows, suggesting that this bacterium may be a part of the normal reproductive tract microbiome (Broes et al. 2019; Kellerman et al. 2022). Additionally, *A. hyovaginalis* was isolated from the tonsils and the nasal conchae of healthy piglets before and after weaning, indicating that it may also be a component of the nasal and tonsillar microbiome (Baele et al. 2001). In wild boars, the source of *A. hyovaginalis* infection is likewise unknown, as there is only one previous report describing this infection in this suid species (Alssahen et al. 2020). Because wild boars and domestic pigs are subspecies of the same species, it is plausible that this agent may also act as a commensal organism within the normal wild boar microbiome, as hypothesized for domestic pigs. As *A. hyovaginalis* is considered a possible commensal organism in wild boars, immunosuppressive factors may have facilitated the development of pneumonia in this case. Porcine circovirus 2 (PCV2) infection, which is frequently detected in Brazilian wild boars (Dal Santo et al. 2022), could have been implicated as an immunosuppressive factor for this wild boar; however, although the available samples were not tested for PCV2 by PCR, infection was considered unlikely, as neither the lymph node nor the lung samples contained histologic lesions consistent with PCV2-associated disease. Likewise, a concurrent *Metastrongylus* spp. infection cannot be completely excluded, since this parasite is frequently reported in Brazilian wild boars (Oliveira et al. 2023) and only selected lung fragments, rather than the entire lungs, were submitted for examination. Pulmonary infection by *Metastrongylus* spp. may predispose animals to secondary bacterial pneumonia, a well-recognized association in domestic pigs (Brewer and Greve 2019). However, neither adult nematodes within the bronchi nor histologic lesions consistent with metastrongylosis were observed in the examined tissues. Despite the severity of the lesions observed in the present case, it seems unlikely that *A. hyovaginalis* represents a significant pathogen transmissible from free-ranging wild boars to domestic pigs; however, further studies are needed to clarify this possibility. Furthermore, *A. hyovaginalis* likely does not pose a zoonotic risk, as there are no reports of human infections caused by this bacterium.

In wild boars, the main differential diagnosis for this pattern of gross and histologic lesions is tuberculosis (Zanella et al. 2008; Lopes et al. 2021b). This differential was considered unlikely due to the presence of Gram-positive bacterial colonies within necrotic areas, the absence of acid-fast bacilli in Ziehl-Neelsen stain, and the absence of regional lymph node involvement (Zanella et al. 2008); however, a co-infection of *A. hyovaginalis* and *Mycobacterium* spp. cannot be totally excluded, as no culture or PCR could be performed. Embolic suppurative bacterial pneumonia, as described in domestic pigs (Piva et al. 2020), mainly associated with *Staphylococcus aureus* infection (Kruse et al. 2015), was also considered a gross differential diagnosis; however, it was excluded histologically because *S. aureus*-induced lesions are typically suppurative (Piva et al. 2020). Excluding both conditions is important because they may represent zoonotic risks for consumers of game meat. Regarding embolic pneumonia in wild boars, only one case has been reported, consisting of suppurative pneumonia caused by bacterial embolism of *Staphylococcus hyicus* in a case of exudative epidermitis (Pérez et al. 2013). Rarely, in domestic pigs, *Actinobacillus pleuropneumoniae* serotype 2 has been associated with granulomatous pleuropneumonia; however, the granulomatous inflammatory response caused by this agent typically exhibits the Splendore-Hoeppli phenomenon (Ohba et al. 2009). Although *Actinomyces*, which was isolated in our case, may also induce the Splendore-Hoeppli phenomenon, this feature was not observed. Additionally, *Rhodococcus equi* infection has been reported to cause pyogranulomatous pneumonia in wild boars (Vargas et al. 2013). Nevertheless, these differential diagnoses were ruled out following the pure culture growth and identification of *A. hyovaginalis*.

This report describes a case of pyogranulomatous pneumonia caused by *A. hyovaginalis* infection in a free-ranging wild boar, highlighting the gross, histopathological, and microbiological findings. Although not previously reported in wild boars, *A. hyovaginalis* infection should be considered a differential diagnosis for pyogranulomatous pulmonary lesions in this suid species, as in domestic pigs.

## Data Availability

No datasets were generated or analysed during the current study.
